# In vitro analysis of antiseptic solution effects on *Staphylococcus aureus* biofilms on orthopedic implant materials

**DOI:** 10.5194/jbji-11-493-2026

**Published:** 2026-08-13

**Authors:** Madison Balagtas, Kenneth Chrulski, Marina Feffer, Matthew Baldridge, Ashley E. Levack

**Affiliations:** 1 Stritch School of Medicine, Loyola University of Chicago, Maywood, Illinois, United States of America; 2 Biostatistics, Clinical Research Office, Stritch School of Medicine, Loyola University Chicago, Maywood, Illinois, United States of America; 3 Department of Orthopaedic Surgery and Rehabilitation, Loyola University Medical Center, Maywood, Illinois, United States of America

## Abstract

Implant-related biofilm infections remain a major challenge in orthopedic surgery. Prior in vitro comparisons have focused on arthroplasty surfaces using static models; fracture fixation implants remain unstudied. We compared six antiseptic solutions against mature *S. aureus* biofilms on stainless steel, the most common fracture fixation metal, using a continuous-flow CDC biofilm reactor, more closely approximating hydrodynamic surgical conditions than static systems. Mature biofilms were created on stainless steel coupons using a continuous-flow reactor for 72 h, then washed for 3 min with one of six irrigation solutions: normal saline, 10 % PI (povidone-iodine), 0.35 % PI, a 10 % PI + 3 % hydrogen peroxide mixture, hypochlorous acid, or 0.05 % chlorhexidine gluconate. Colony-forming units (CFUs) were quantified following standardized sonication and plating, and mixed-effects negative binomial regression was used to estimate treatment effects. All antiseptic solutions produced statistically significant reductions in biofilm-associated CFUs relative to untreated controls. Ten percent PI demonstrated the greatest reduction (3.46- ${\mathrm{log}}_{10}$ ), with 0.35 % PI producing a similar 3.42- ${\mathrm{log}}_{10}$ reduction. The PI–hydrogen peroxide mixture achieved a 3.24- ${\mathrm{log}}_{10}$ reduction without evidence of synergistic activity. Hypochlorous acid and chlorhexidine produced 2.95- ${\mathrm{log}}_{10}$ and 2.46- ${\mathrm{log}}_{10}$ reductions, respectively, while saline demonstrated minimal effect. These findings indicate that PI solutions, even at dilute concentrations, are highly effective at disrupting established biofilms on stainless steel. As an in vitro single-organism model, these findings establish a comparative efficacy baseline on a fracture-fixation-relevant surface but require validation in multispecies, multi-material, and mechanically realistic irrigation systems before clinical translation.

## 1 Introduction

Implant-related bacterial biofilm infections remain a current challenge in orthopedic surgery (Whitley et al., 2022). Biofilms, especially those formed by staphylococcal species, lead to chronic infections and pose difficulties in treatment (Arciola et al., 2018). These structured, surface-adherent microbial communities are encased within an extracellular polymeric matrix composed of polysaccharides, proteins, extracellular DNA, and other biomolecules (Flemming et al., 2016). Structural functional dynamics of *Staphylococcus aureus* biofilms and biofilm matrix proteins on different clinical materials have been extensively studied, revealing the complex interactions between bacterial adhesion and implant surface properties (Hiltunen et al., 2019).

*Staphylococcus aureus* is a predominant pathogen in orthopedic implant infections, demonstrating remarkable virulence and adaptability (Tong et al., 2015). Bacteria living in biofilms can exhibit 10- to 1000-fold increased antibiotic tolerance compared to their planktonic counterparts (Mah and O'Toole, 2001). This tolerance arises through multiple synergistic mechanisms, including limited antibiotic penetration through the extracellular matrix, metabolically dormant persistent cells in deeper biofilm layers, and altered microenvironments that protect bacteria from antimicrobial agents (Stewart and Costerton, 2001).

The current management of implant-associated infection typically involves surgical debridement combined with systemic antibiotic therapy (Depypere et al., 2020). Depending on the chronicity of infection, implant retention can sometimes be considered. Consequently, intraoperative irrigation with antiseptic solutions has emerged as an important adjuvant to surgical debridement to supplement the mechanical removal of contaminated tissue and chemically reduce bacterial bioburden. Various antiseptic agents are routinely employed in orthopedic practice, including povidone-iodine, hydrogen peroxide, hypochlorous acid, and chlorhexidine-based solutions (Chao et al., 2025). Despite the widespread clinical adoption of antiseptic irrigation, comparative data on the efficacy against established biofilms on orthopedic implant materials remain limited. Several prior in vitro investigations have compared antiseptic solutions against orthopedic biofilms, predominantly using static well-plate models on arthroplasty surfaces, including porous titanium, polymethylmethacrylate, cobalt-chromium, and oxidized zirconium (Premkumar et al., 2021; Chao et al., 2025; Hamad et al., 2025; Schwechter et al., 2011). Stainless steel has been used as a biofilm substrate in prior in vitro investigations of surface modification strategies (Akens et al., 2018); however, its susceptibility to antiseptic irrigation has not been directly studied. Collectively, prior work demonstrates consistent efficacy for 10 % povidone-iodine; however, results for dilute PI and chlorhexidine vary considerably across surface types and biofilm models. Notably, Chao et al. (2025) found that 0.35 % PI and 0.05 % CHG failed to achieve a 3- log⁡ reduction against mature MSSA biofilm on cobalt-chromium and oxidized zirconium using a static incubation model. Whether these findings extend to stainless steel, the most widely used metal in fracture fixation hardware, and to biofilms grown under continuous hydrodynamic flow conditions as produced by the CDC biofilm reactor, has not been established.

Biofilm formation occurs through stages: initial reversible attachment, irreversible anchoring via surface adhesions, microcolony formation, maturation into a three-dimensional architecture, and eventual dispersal of planktonic cells (Flemming et al., 2016; O'Toole et al., 2000). Mature biofilms exhibit substantially greater tolerance to irrigation than planktonic bacteria, and volume-based dilution alone is insufficient against EPS-encased organisms (Mah and O'Toole, 2001; Stewart and Costerton, 2001). This motivates the use of antiseptic agents with direct antibiofilm activity.

The purpose of this study was to evaluate and compare the antibiofilm efficacy of six commonly used antiseptic irrigation solutions against *S. aureus* biofilms cultured on stainless steel coupons under continuous-flow conditions using the CDC biofilm reactor. We hypothesized that povidone-iodine solutions would demonstrate superior antibiofilm activity compared to other commercially available products. The findings extend prior arthroplasty-focused comparisons to a fracture-fixation-relevant surface and model, providing a foundational evidence base for future investigation.

## 2 Methods

### 2.1 Bacterial strain and culture preparation

Methicillin-sensitive *S. aureus* (MSSA) (ATCC 49230) was obtained from the American Type Culture Collection and maintained in frozen glycerol stocks at -80 °C. An overnight culture was prepared by streaking from the glycerol stock onto a tryptic soy broth (TSB) agar plate, incubating at 37 °C overnight, and inoculating a single isolated colony into 5 mL of TSB in a 15 mL conical tube. The tube was incubated at 37 °C with continuous shaking at 250 rpm for 18–24 h. The overnight culture was centrifuged at 3000 rpm for 10 min at 25 °C, and the bacterial pellet was washed three times with sterile phosphate-buffered saline (PBS) (10 mL per wash) to remove residual media. The pellet was resuspended in 10 mL of fresh TSB and placed immediately on ice. OD600 was measured against a plain TSB blank at 600 nm. If OD600 was below 0.490, the suspension was briefly reincubated at 37 °C with shaking in 3–5 min increments until the target of 0.490–0.510 (corresponding to approximately 10^8^ CFU mL^−1^) was reached. Serial dilutions and spread plating onto TSB agar were performed immediately prior to reactor inoculation to confirm the bacterial concentration.

### 2.2 Biofilm formation using CDC biofilm reactor

**Figure 1:**
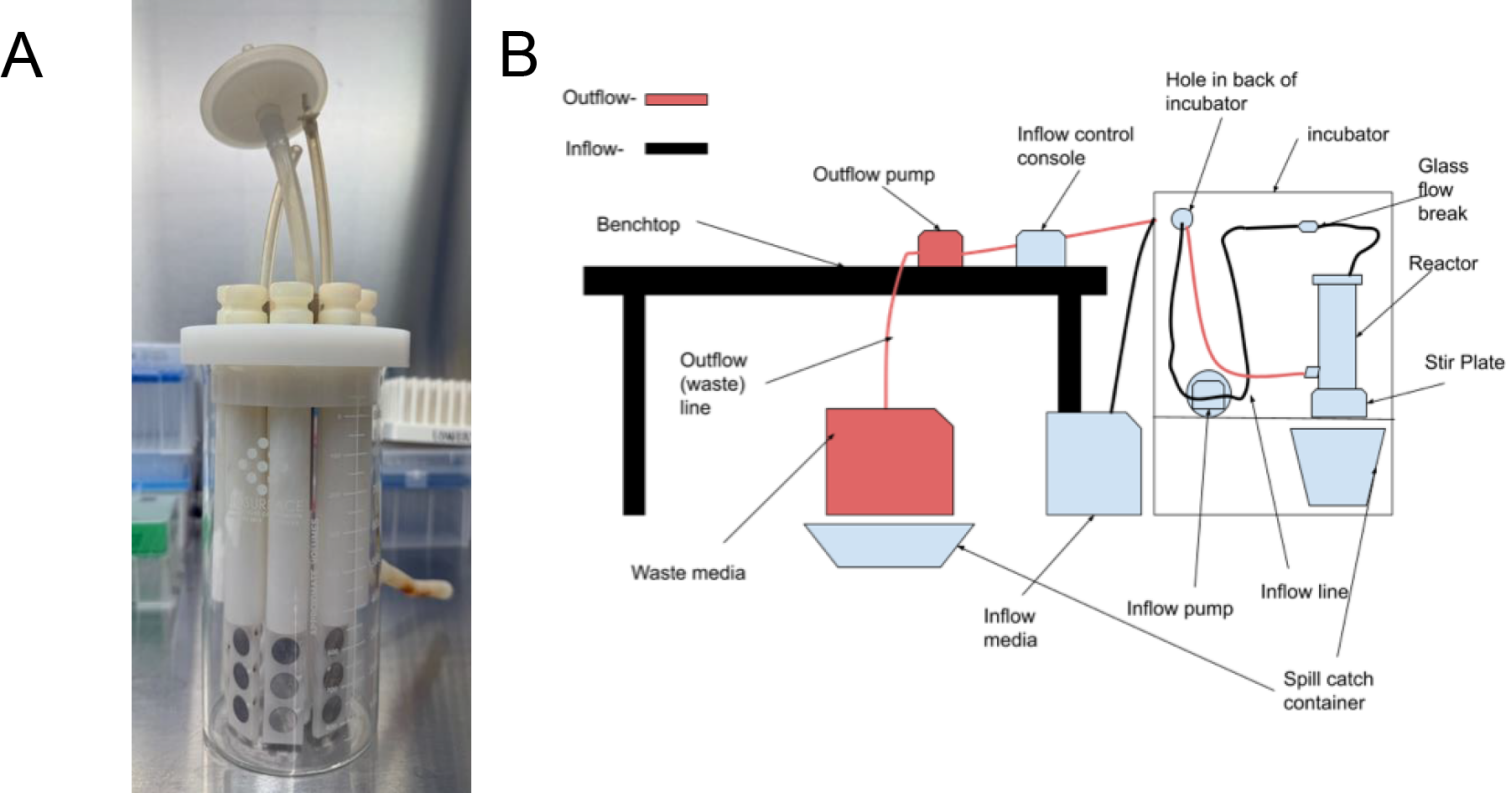
CDC biofilm reactor setup. **(A)** Photograph of the CDC biofilm reactor vessel showing stainless steel disk coupons mounted in polypropylene rods during biofilm growth. **(B)** Schematic diagram of the complete experimental setup. Media flows from the inflow carboy through the peristaltic inflow pump (7.2 rpm) into the glass flow break, then into the CDC reactor vessel containing stainless steel coupons mounted in polypropylene rods (70 rpm magnetic stir). Effluent exits through the outflow pump into the waste carboy. The entire system was housed in a 37 °C incubator throughout biofilm cultivation.

Biofilms were cultivated using a CDC Biofilm Reactor (BioSurface Technologies, Bozeman, MT), a validated system for growing reproducible biofilms under controlled hydrodynamic conditions (Kay et al., 2022; see Fig. 1). Sterile 316L stainless-steel disk-shaped coupons (10 mm diameter; BioSurface Technologies), sterilized by autoclaving, were mounted in polypropylene coupon holders and inserted into the reactor vessel containing 500 mL of TSB. The 316L alloy and 10 mm disk format are consistent with prior in vitro stainless steel biofilm studies (Akens et al., 2018). A representative photograph of the coupons is provided in Fig. S1 in the Supplement.

For inoculation, a 1:10 dilution of the OD-adjusted bacterial suspension was prepared (100 µ L into 900 µ L TSB, yielding approximately 10^7^ CFU mL^−1^), and 1 mL of this diluted suspension was introduced into the reactor via the inoculation port, yielding a starting concentration of approximately 10^5^ CFU mL^−1^ in 500 mL TSB, consistent with prior stainless steel biofilm studies (Akens et al., 2018). The reactor was operated in batch mode for 24 h at 37 °C with stirring at 70 rpm and the outflow tube clamped shut. Following the 24 h batch phase, the reactor was switched to continuous-flow mode for 48 h. During the flow phase, 10 % TSB (1 L TSB in 9 L autoclaved milliQ water) was supplied via peristaltic pump at 6.94 mL min^−1^ (pump speed 7.2 rpm), delivering approximately 20 L over 48 h from two 10 L carboys refreshed every 24 h. Stirring was maintained at 70 rpm throughout. Total biofilm cultivation time was 72 h (24 h batch phase plus 48 h flow phase). The reactor was maintained at 37 °C throughout.

### 2.3 Antiseptic solution treatment

After 72 h of total growth time, the coupons were aseptically removed from the reactor using sterile forceps and gently rinsed in 10 mL of sterile PBS to remove non-adherent planktonic bacteria. Each coupon was then placed into a separate well of a sterile six-well tissue culture plate containing 6 mL of one of the following test solutions:

1. *Untreated control* (coupons removed from reactor without antiseptic exposure),
2. *Normal saline* (0.9 % sodium chloride),
3. *10 % povidone-iodine*,
4. *0.35 % povidone-iodine* (diluted from 10 % stock with sterile saline),
5. *10 % povidone-iodine* + *3 % hydrogen peroxide* ( 1:1 mixture),
6. *Vashe wound solution* (hypochlorous acid),
7. *Irrisept* (0.05 % chlorhexidine gluconate (CHG) irrigation).

The 3 min dwell time was selected to reflect established clinical orthopedic irrigation protocols (Brown et al., 2012; Goswami and Austin, 2019) and to enable direct comparison with prior biofilm efficacy studies using identical conditions (Premkumar et al., 2021; Chao et al., 2025). An untreated control group (coupons removed from reactor and processed without antiseptic exposure) and a normal saline group were included in each experimental run to distinguish the effects of the mechanical rinsing procedure from the chemical antibiofilm activity of each antiseptic solution. A volume of 6 mL per well was used to fully submerge each coupon, ensuring complete antiseptic contact with all biofilm-bearing surfaces. The 0.35 % PI concentration was chosen as the most widely adopted dilute PI formulation in intraoperative irrigation practice (Brown et al., 2012). The simultaneous 1:1 PI-H_2_O_2_ mixture was selected to assess a preparation strategy used in some clinical centers, contrasting with the sequential protocol of Wan et al. (2025) evaluated in the Discussion. Commercial products were tested at manufacturer-recommended concentrations.

### 2.4 Biofilm disruption and CFU

**Figure 2:**
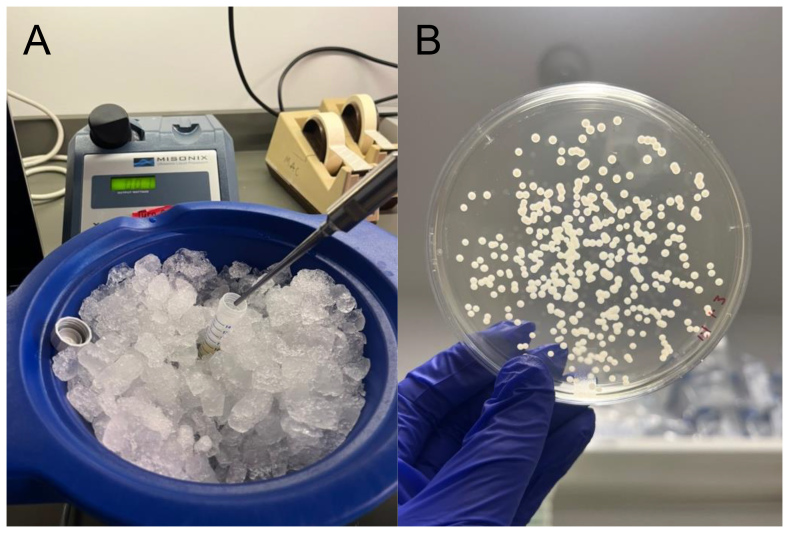
Biofilm disruption and CFU enumeration workflow. **(A)** Photograph of the sonication step used to dislodge bacteria from biofilm-coated coupons prior to quantification. **(B)** Representative agar plate showing bacterial colonies following serial dilution and spread plating for colony-forming unit (CFU) determination.

Following antiseptic treatment, coupons were rinsed three times in PBS by gentle submersion in a PBS wash reservoir (approximately 2–3 s per wash) to remove residual antiseptic solution and non-adherent bacteria. Each coupon was transferred to a 15 mL conical tube containing 10 mL of fresh TSB. Biofilm-associated bacteria were recovered by probe sonication using a Misonix XL-2000 Series ultrasonic liquid processor at approximately 17–18 W output, using a manual pulse technique of 5 s on and 5 s off for a cumulative active sonication time of 5 min (see Fig. 2). Pulsing was performed manually by the operator monitoring a countdown timer throughout the procedure; the automated pulse function of the instrument was not used. Samples were maintained on ice throughout sonication to prevent thermal inactivation of bacteria. The sonicator probe was decontaminated with 10 % bleach followed by 70 % ethanol between each sample. Following sonication, each tube was vortexed for 10 s to ensure uniform suspension of biofilm-derived bacteria.

A 10-fold serial dilution series was prepared from the sonicated suspension (100 µ L into 900 µ L TSB, repeated to 10^−5^). Prior to data collection runs, an initial optimization experiment using spot plating (10 µ L per dilution spotted in triplicate onto TSB agar) was performed to determine the optimal dilution range for each treatment condition. All subsequent data collection was performed by spread plating 50 µ L of the appropriate dilution onto TSB agar in triplicate. Plates were incubated at 37 °C for 18–24 h, after which colonies were manually counted. CFU mL^−1^ was calculated as average colony count × 20 × dilution factor. Positive controls (untreated biofilm coupons processed identically) were included in each experimental run.

Each antiseptic treatment condition was tested using three independent coupons per experiment. The entire experimental protocol (bacterial culture through CFU enumeration) was repeated on three separate occasions ( n=3 independent experiments) to assess reproducibility and to account for potential day-to-day variability. All manipulations were performed under aseptic conditions in a laminar flow biosafety cabinet.

### 2.5 Statistical analysis

Varying irrigation solution types were assessed for ability to reduce colony-forming units (CFUs) among plates of *S. aureus* compared to a control type. Four experimental runs were completed on separate dates. Per run, three coupons were assigned to each of the seven treatments, with three agar plates examined per coupon, for a total of 252 plates across all runs and treatment groups. Bacterial counts were modeled using a mixed-effects negative binomial regression model to account for the overdispersed count distribution of biofilm-associated CFUs and the clustering of culture plates within experimental run dates. Fixed effects were specified for solution type and experimental run date; random effects were specified for culture plate. A three-level hierarchical model incorporating coupon-level clustering was also evaluated; however, the coupon-level variance estimate was negligible, and model-fit criteria including AIC, BIC, and log-likelihood showed no improvement over the two-level model. As the higher-level random effect did not improve fit or change substantive conclusions relative to the two-level model, the more parsimonious two-level specification was therefore retained as the most interpretable representation of the data. An unstructured covariance matrix was specified to avoid imposing restrictive assumptions on the random-effects variance-covariance structure. Robust (sandwich) standard errors were used to ensure valid inference under potential heteroskedasticity or mild model misspecification. Estimates of mixed negative binomial regression were used to determine estimated mean CFUs and log reductions for each solution type examined. Estimates of mean CFUs are displayed with corresponding p values, testing whether the estimate significantly differs from zero, while log reductions are displayed as pair-wise comparisons, with p values denoting significant differences from the referent group, “untreated control”. Given that all primary effects were significant at p<0.001 , well below any conventional multiplicity-adjusted threshold, formal multiplicity correction does not alter the interpretation of results and was not applied. All predicted CFU values and log reductions reported are model-derived estimates with 95 % confidence intervals. All tests are two-sided with a threshold for significance of p<0.05 . All analyses were completed via Stata v.18 (StataCorp. College Station, TX).

## 3 Results

Using the CDC biofilm reactor system, *S. aureus* biofilms were grown on all stainless steel coupons over the 72 h period. The results depicted in Table 1 show that the control group, consisting of biofilm-coated coupons removed from the reactor without antiseptic exposure, produced a mean bacterial burden of $6757.3\times 10^{3}$ CFU mL^−1^ (95 % CI: 4969.1–8545.5).

**Table 1:** Predicted mean CFUs per irrigation type, adjusted for date of experiment and dependence within coupon.

Solution type	Predicted mean CFU 10^3^	p value^*^
	per mL (95 % CI)	
Control	6757.3 (4969.1, 8545.5)	<0.001
Saline	4758.7 (3119.2, 6398.2)	<0.001
10 % PI	2.3 (1.1, 3.5)	<0.001
0.35 % PI	2.5 (0.6, 4.5)	0.011
10 % PI: 3 % HP	3.9 (1.5, 6.2)	0.001
HA	7.5 (3.1, 12.0)	0.001
CHG	23.2 (1.1, 45.3)	0.040

^*^ p values associated with hypothesis that predicted mean is not equal to zero.

After adjusting for experimental date effects and accounting for dependence within coupons in the mixed-effects negative binomial regression model, all tested antiseptic solutions demonstrated statistically significant antibiofilm activity compared to the untreated control (Table 1). The rank order of efficacy based on predicted mean CFU counts was as follows (presented in increasing magnitude of viable bacteria remaining on the coupons following coupon exposure to the irrigant solution): 10 % povidone-iodine ( $2.3\times 10^{3}$ CFU mL^−1^; 95 % CI: 1.1–3.5) > 0.35 % povidone-iodine ( $2.5\times 10^{3}$ CFU mL^−1^; 95 % CI: 0.6–4.5) > 10 % povidone-iodine + 3 % hydrogen peroxide ( $3.9\times 10^{3}$ CFU mL^−1^; 95 % CI: 1.5–6.2) > hypochlorous acid solution ( $7.5\times 10^{3}$ CFU mL^−1^; 95 % CI: 3.1–12.0) > chlorhexidine gluconate ( $23.2\times 10^{3}$ CFU mL^−1^; 95 % CI: 1.1–45.3) > normal saline ( $4758.7\times 10^{3}$ CFU mL^−1^; 95 % CI: 3119.2–6398.2).

The 10 % povidone-iodine solution exhibited the greatest antibiofilm efficacy, achieving a 3.46- ${\mathrm{log}}_{10}$ reduction (99.97 % reduction) in viable bacteria compared to the untreated control (incidence rate ratio (IRR) = 0.0003; 95 % CI: 0.0002–0.0006; p<0.001 ). This means that coupons treated with 10 % povidone-iodine had only 0.03 % of the bacterial burden compared to untreated controls. The dilute 0.35 % povidone-iodine solution demonstrated nearly equivalent performance with a 3.42- ${\mathrm{log}}_{10}$ reduction (99.96 % reduction; IRR = 0.0004; 95 % CI: 0.0002–0.0008; p<0.001 ). The difference in log reduction between 10 % and 0.35 % concentrations was minimal (0.04 logs), and the confidence intervals for these two solutions overlapped substantially. Both concentrations surpassed the predefined antibiofilm efficacy benchmark of 3- ${\mathrm{log}}_{10}$ reduction, which has been used as an operational efficacy threshold in prior orthopedic biofilm studies (Premkumar et al., 2021; Chao et al., 2025). All comparisons are made against the untreated control group. This threshold does not represent a clinically validated cut-off for surgical infection prevention and should not be interpreted as directly predicting clinical outcomes (Table 2).

**Table 2:** Antiseptic solution efficacy against MSSA biofilm on stainless steel: log reduction and incidence rate ratios compared to untreated control.

Solution	% CFU	Log reduction	IRR (95 % CI)	p value
type	reduced	in CFUs		
	compared	compared to		
	to control	control		
Control				
Saline	29.58 %	0.15	0.7042 (0.5039, 0.9843)	0.034
PI	99.97 %	3.46	0.0003 (0.0002, 0.0006)	<0.001
0.35 % PI	99.96%	3.42	0.0004 (0.0002, 0.0008)	<0.001
1:1	99.94 %	3.24	0.0006 (0.0003, 0.0011)	<0.001
HA	99.89 %	2.95	0.0011 (0.0006, 0.0020)	<0.001
CHG	99.67 %	2.46	0.0034 (0.0015, 0.0081)	<0.001

The 1:1 mixture of 10 % povidone-iodine with 3 % hydrogen peroxide produced a 3.24- ${\mathrm{log}}_{10}$ reduction (99.94 % reduction) in biofilm-associated bacteria (IRR = 0.0006; 95 % CI: 0.0003–0.0011; p<0.001 ). While this combination also exceeded the 3-log threshold, it demonstrated slightly lower efficacy compared to povidone-iodine alone, with reductions of 0.22 logs and 0.18 logs less than the 10 % and 0.35 % povidone-iodine solutions, respectively. The incidence rate ratio indicated that coupons treated with the combination solution retained approximately twice the bacterial burden (IRR = 0.0006) compared to 10 % povidone-iodine alone (IRR = 0.0003), although both achieved greater than 99.9 % bacterial reduction. No synergistic enhancement was observed with the addition of hydrogen peroxide to povidone-iodine (Table 2).

Vashe hypochlorous acid wound solution achieved a 2.95- ${\mathrm{log}}_{10}$ reduction (99.89 % reduction) in bacterial CFU counts compared to the control (IRR = 0.0011; 95 % CI: 0.0006–0.0020; p<0.001 ). Irrisept chlorhexidine gluconate lavage demonstrated a 2.46- ${\mathrm{log}}_{10}$ reduction (99.67 % reduction; IRR = 0.0034; 95 % CI: 0.0015–0.0081; p<0.001 ). While both commercial products exhibited greater than 99 % bacterial killing, neither reached the 3- ${\mathrm{log}}_{10}$ reduction threshold. Irrisept yielded approximately 10-fold higher residual bacterial counts ( $23.2\times 10^{3}$ CFU mL^−1^) compared to the povidone-iodine solutions (2.3– $3.9\times 10^{3}$ CFU mL^−1^), as reflected in its higher incidence rate ratio (Table 2).

Normal saline irrigation produced a statistically significant but clinically modest reduction in biofilm bacteria, with only a 0.15- ${\mathrm{log}}_{10}$ reduction (29.58 % reduction) compared to the untreated control (IRR = 0.7042; 95 % CI: 0.5039–0.9843; p=0.034 ). The mean CFU count following saline treatment ( $4758.7\times 10^{3}$ CFU mL^−1^) remained substantially elevated and within the same order of magnitude as the untreated control ( $6757.3\times 10^{3}$ CFU mL^−1^). The IRR of 0.70 indicates that saline treatment reduced bacterial burden by only approximately 30 % (Table 2).

The mixed-effects negative binomial regression model demonstrated appropriate fit to the count data, with incidence rate ratios (IRRs) providing interpretable effect sizes for clinical translation. For example, the IRR of 0.0003 for 10 % povidone-iodine indicates that for every 10 000 CFUs present on an untreated control coupon, only three CFUs would be expected on a coupon treated with 10 % povidone-iodine, holding the experimental date constant. Adjustment for experimental date had minimal impact on treatment effect estimates, indicating consistent results across independent experimental runs (Table 2).

## 4 Discussion

In this in vitro study, we demonstrate that povidone-iodine-based irrigation solutions (both 10 % and dilute 0.35 %) achieved the greatest reduction in biofilm-associated *S. aureus* burden on stainless steel. Both concentrations exceeded the accepted ≥ 3- ${\mathrm{log}}_{10}$ reduction threshold for bactericidal antibiofilm activity. This is clinically relevant, as many surgeons dilute 10 % PI out of concern for cytotoxicity to osteoblasts and soft tissue. Our results align with prior work by Oduwole et al. (2010), who showed that even sub-inhibitory PI concentrations (0.17 %, 0.35 %, and 0.7 %) significantly inhibited *S. epidermidis* and *S. aureus* biofilm formation through downregulation of the icaADBC operon. Taken together, these complementary data support PI's antibiofilm properties across sub-inhibitory PI concentration ranges and reinforce its value as an irrigation solution capable of both preventing biofilm formation and eradicating established biofilm on implant-grade materials.

These findings diverge from Chao et al. (2025), who used a static well-plate biofilm model and found that 0.35 % PI and 0.05 % CHG both failed to achieve a 3- log⁡ reduction against mature MSSA biofilm on cobalt-chromium and oxidized zirconium arthroplasty surfaces. In the present study, both 0.35 % PI and CHG produced statistically significant reductions on stainless steel under continuous-flow conditions, with 0.35 % PI achieving a 3.42- log⁡ reduction comparable to 10 % PI. We hypothesize that biofilms grown in the CDC continuous-flow reactor may differ structurally from static well-plate biofilms which could improve antiseptic penetration. This hypothesis is consistent with the observation by Akens et al. (2018) that biofilm architecture on stainless steel varies with incubation conditions. Differences in coupon surface material and MSSA strain (ATCC 49230 vs. Xen36) may also contribute. These model-dependent differences underscore the importance of testing multiple biofilm systems and surface.

The antibiofilm mechanisms of the tested solutions differ. Povidone-iodine releases free molecular iodine (I2), which oxidizes microbial membrane lipids, proteins, and nucleic acids; dilute formulations may release a higher proportion of free I2 relative to total iodine content, which could partly explain the preserved efficacy observed at 0.35 % (Wang et al., 2022). Chlorhexidine gluconate disrupts bacterial membranes via electrostatic binding to phospholipids. In vitro evidence demonstrates that PI at concentrations of 0.1 % and above exerts cytotoxic effects on osteoblasts, fibroblasts, and myoblasts (Liu et al., 2017; Wang et al., 2022), although clinical series have not consistently found adverse wound healing outcomes at concentrations used intraoperatively (Brown et al., 2012). Future work combining antibiofilm efficacy with cytotoxicity profiling in the same experimental model would strengthen evidence-based antiseptic selection.

This study used a static 3 min soak to isolate chemical antibiofilm activity under controlled conditions. Intraoperative irrigation involves additional mechanical forces from high-volume lavage or pulse lavage systems that contribute to bacterial debridement independent of antiseptic chemistry. Schwechter et al. (2011) demonstrated that pulse lavage combined with antiseptic irrigation reduced bacterial burden better than antiseptic exposure alone in an implant biofilm model. The static soak design likely underestimates total antibiofilm effect achievable intraoperatively, while allowing for controlled comparison of chemical efficacy across solutions. Future studies incorporating pulsatile flow and clinically representative irrigation volumes will improve translational applicability.

The FRI Consensus Group recommends that intraoperative irrigation in fracture-related infections be performed with normal saline at low pressure, noting that the use of antiseptic additives is currently not advised due to concerns about potential cytotoxicity (Metsemakers et al., 2020). The present study provides in vitro data relevant to this ongoing clinical discussion. Our findings demonstrate that several antiseptic solutions, including 0.35 % PI, achieve significant reductions in established MSSA biofilm on stainless steel, the predominant fracture fixation material, under controlled laboratory conditions. While these results cannot be directly extrapolated to the intraoperative setting, and the cytotoxicity concerns that underpin the current recommendation are acknowledged as an important limitation of this work, they contribute to the growing body of in vitro evidence that may inform future clinical evaluation of antiseptic additives in FRI debridement.

No synergistic benefit was observed by combining povidone-iodine with hydrogen peroxide. Although the mixture achieved > 3- log⁡ reduction, performance was slightly inferior to PI alone. This does not align with prior studies, suggesting that combining antiseptics enhances efficacy. A likely explanation may be due to methodological differences from Wan et al. (2025), who also used a biofilm model but applied their combination sequentially: treating biofilm with 3.5 % HP for 5 min followed by 1 % PI for an additional 5 min, rather than exposing biofilm to a mixed solution. As a result, their combination arm had double the total contact time relative to other treatment groups. These procedural differences in concentration, sequence, and total exposure time may explain why synergy was detected in their system but not in ours, despite both studies evaluating biofilm-embedded bacteria.

Hypochlorous acid (Vashe) and chlorhexidine gluconate (Irrisept) both demonstrated significant antibiofilm effects ( >2 - log⁡ reduction) but did not meet the ≥3 - log⁡ threshold. These findings parallel emerging literature showing that not all commercially marketed irrigation solutions used in orthopedic practice provide true bactericidal antibiofilm activity. Normal saline achieved only a minimal effect, confirming that saline alone is insufficient against mature biofilms. Evaluating saline under conditions that include mechanical disruption, such as pulse lavage, represents an important direction for future study.

Taken together, these findings reinforce the fact that povidone-iodine solutions remain highly effective for established *S. aureus* biofilms and support its continued consideration in orthopedic infection management.

This study has limitations. This was an in vitro bench model. Although the CDC reactor is validated and widely used for orthopedic biofilm research, it does not fully replicate in vivo complexity. Second, only MSSA was evaluated. Biofilm behavior and antiseptic susceptibility vary across organisms, including methicillin-resistant *S. aureus* (MRSA), coagulase-negative staphylococci, gram negatives, and polymicrobial states typical of chronic implant infections. Third, we utilized a 3 min dwell period; operative dwell times vary widely, and irrigation can be pulsatile, sequential, and/or prolonged. CFU counting was performed by a single investigator with knowledge of treatment group assignment; formal blinding was not employed. Fourth, only stainless steel coupons were tested, and multiple factors influence bacterial adhesion and antiseptic penetration. Finally, cytotoxicity to bone or soft tissue was not evaluated, which is clinically relevant, as surgeons must balance bacterial killing with preservation of host cell viability.

## 5 Conclusion

In this in vitro CDC reactor model of MSSA biofilm on stainless steel, povidone-iodine solutions (both 10 % and 0.35 %) demonstrated the greatest antibiofilm activity, each achieving ≥3 - ${\mathrm{log}}_{10}$ reductions in viable bacteria. Hypochlorous acid and chlorhexidine gluconate demonstrated significant but sub-threshold reductions, and saline alone provided minimal effect.

These findings provide additional in vitro evidence supporting the antibiofilm activity of povidone-iodine, and justify further translational and clinical investigation of its role in intraoperative irrigation for orthopedic procedures. Future work should evaluate multiple species, clinically realistic irrigation workflows, and cytotoxicity profiles across implant materials to better inform clinical translation.

## Supplement

10.5194/jbji-11-493-2026-supplement The supplement related to this article is available online at https://doi.org/10.5194/jbji-11-493-2026-supplement.

## Data Availability

The datasets and statistical syntax generated during the study are available from the corresponding author upon reasonable request.

## Code availability

This study did not generate custom software code (e.g., no script-based workflow). Statistical analyses were performed using the proprietary third-party software Stata (version 18; StataCorp LLC, College Station, TX), which is available via a commercial license from the vendor. Therefore, no underlying source code is publicly accessible.
