# Supplementary material for: In vitro analysis of antiseptic solution effects on Staphylococcus aureus biofilms on orthopedic implant materials
Source: J Bone Jt Infect. 2026 Aug 13;11(4):493–501. doi: 10.5194/jbji-11-493-2026 (PMC13479933; doi:10.5194/jbji-11-493-2026)
Supplement: Supplementary file 1 [file jbji-11-493-2026-supplement.pdf]

Supplement of J. Bone Joint Infect., 11, 493–501, 2026
https://doi.org/10.5194/jbji-11-493-2026-supplement
© Author(s) 2026. CC BY 4.0 License.

Supplement of

In vitro analysis of antiseptic solution effects on *Staphylococcus aureus* biofilms on orthopedic implant materials

Madison Balagtas et al.

Correspondence to: Madison Balagtas (mbalagtas@luc.edu)

The copyright of individual parts of the supplement might differ from the article licence.

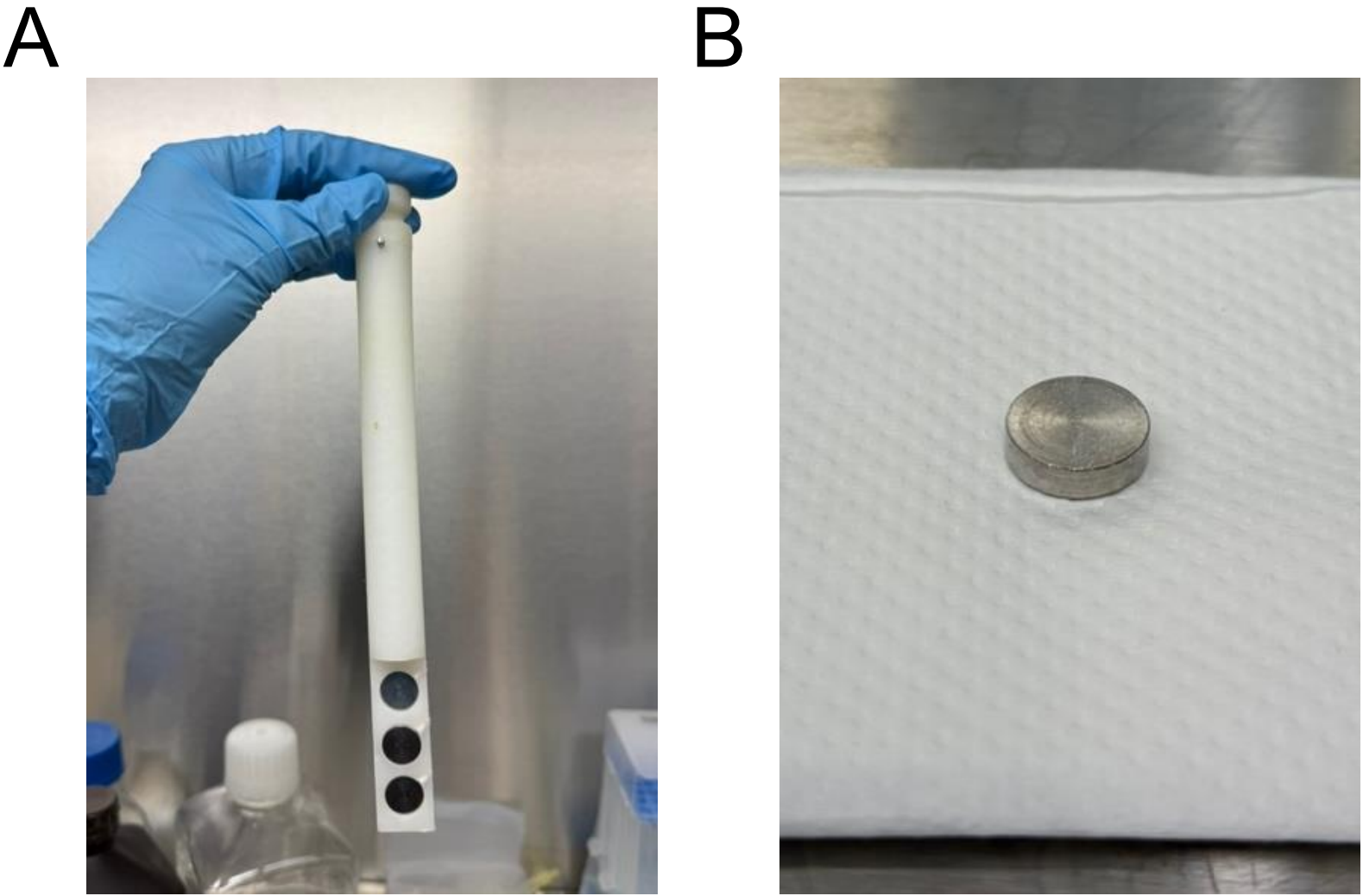

Figure S1. Stainless steel coupons used in biofilem experiments.
(A) Coupon holder containing stainless steel coupons used in the CDC biofilm reactor. (B) Representative stainless steel coupon following removal from the reactor system. Coupons served as the substrate for MSSA biofilm formation and subsequent antiseptic exposure experiments.
